# Prevalence and Antimicrobial Resistance of *Pseudomonas aeruginosa* Recovered from Respiratory and Urinary Specimens in a Lebanese Hospital (2020–2025)

**DOI:** 10.3390/idr18040087

**Published:** 2026-08-13

**Authors:** Rana Barakat, Zeinab Ezzeddine, Rana El Haidari, Samara Hassan, Ghassan Ghssein

**Affiliations:** 1 Laboratory Sciences Department, Faculty of Public Health, Islamic University of Lebanon, Khalde P.O. Box 30014, Lebanon; ranahaidari14@hotmail.com (R.E.H.); sam_hassan77@hotmail.com (S.H.); 2 Faculty of Sciences, Lebanese University, Beirut P.O. Box 6573, Lebanon; ghassan.ghssein@iul.edu.lb; 3 High Council for Scientific Research & Publication (HCSRP), Islamic University of Lebanon, Khalde P.O. Box 30014, Lebanon; 4 Faculty of Public Health, Lebanese University, Hadath P.O. Box 6573/14, Lebanon; 5 National Institute of Public Health, Clinical Epidemiology and Toxicology-Lebanon (INSPECT-LB), Beirut, Lebanon; 6 Laboratory Department, Sibline Governmental Hospital, Sibline, Lebanon

**Keywords:** respiratory tract infections, urinary tract infections, antimicrobial resistance, *Pseudomonas aeruginosa*, Lebanon

## Abstract

Background: Respiratory tract infections (RTIs) and urinary tract infections (UTIs) are among the most common infections in humans. It is estimated that millions of deaths occur each year worldwide due to these infections. *Pseudomonas aeruginosa* (*P. aeruginosa*), in particular, represents a public health threat since it has a high ability to acquire resistance to different antibiotic classes. The aim of this study was to determine the prevalence and antimicrobial resistance patterns of *P. aeruginosa* isolates from patients with RTIs and UTIs in Lebanon. Methods: This retrospective study was performed during 2020–2022 and 2024–2025. Samples were collected from both inpatients and outpatients, where identification and isolation of bacteria were performed along with antibiotic susceptibility testing. Results: The overall prevalence of *P. aeruginosa* isolates in RTI cases over the study period was 10.9%, and that of UTI cases was 1.3%. The general antimicrobial resistance profile of *P. aeruginosa* isolates from RTI and UTI cases showed the highest resistance for Piperacillin–Tazobactam and Aztreonam, respectively. For RTIs, antibiotic resistance decreased from 48.1% in 2020 to 36.7% in 2025 and increased from 16.7% to 19.4% for UTIs. Conclusions: According to our research, *P. aeruginosa* with low antibiotic susceptibility is frequently found in isolated samples, highlighting the significant challenge in treating severe infectious illnesses. To choose focused and efficient antibiotic therapy for RTIs and UTIs and to stop the emergence of multidrug-resistant (MDR) bacteria, it is essential to accurately identify the pathogenic microorganisms and their antibiotic susceptibility patterns.

## 1. Introduction

*Pseudomonas aeruginosa* is an opportunistic pathogenic bacterium that can cause both community and hospital-acquired infections (HAIs), typically in people with preexisting illnesses such as cystic fibrosis, diabetes, or immunosuppression [1,2]. It can cause several types of HAIs, including bloodstream, surgical site, burn wound, respiratory, and urinary tract infections [3].

While Enterobacteriaceae remain the primary cause of uncomplicated urinary tract infections (UTIs), *P. aeruginosa* is a formidable opportunistic pathogen predominantly implicated in healthcare-associated and catheter-associated UTIs (CAUTIs) [4,5]. The pathogenesis of *P. aeruginosa* in complicated UTIs is driven by its rich arsenal of virulence factors—including flagella, pili, alginate production, and biofilm formation on indwelling catheters—which facilitate tissue adherence and persistence against host immune defenses [6,7]. Furthermore, prior broad-spectrum antibiotic exposure creates a strong selective pressure that favors *P. aeruginosa* colonization and infection in high-risk, hospitalized patients [8,9]. Understanding its distinct pathogenicity in complicated urinary tract environments is critical for guiding effective therapeutic and stewardship strategies [10,11].

Furthermore, *P. aeruginosa* is linked to high rates of morbidity and mortality and is a major cause of respiratory tract infections (RTIs), particularly in hospitalized and immunocompromised individuals [12]. Owing to several factors—including its widespread environmental distribution [13,14], frequent multidrug resistance [15,16], and an extensive array of virulence factors—it can cause both acute and chronic respiratory infections. However, establishing a definitive diagnosis of respiratory infection presents a distinct clinical challenge; the recovery of *P. aeruginosa* from a respiratory specimen does not inherently indicate active, tissue-invasive disease, as this organism frequently colonizes the upper airways and tracheobronchial tree without causing acute pathology. Differentiating true respiratory infection—characterized by acute clinical deterioration, systemic inflammatory responses, and diagnostic radiographic changes—from simple asymptomatic airway colonization is therefore essential for accurate epidemiological surveillance and targeted antimicrobial therapy.

*P. aeruginosa* is recognized among the six major multidrug-resistant (MDR) pathogens, alongside *E. coli*, *Staphylococcus aureus* (*S. aureus*), *K. pneumoniae*, *Streptococcus pneumoniae* (*S. pneumoniae*), and *Acinetobacter baumannii* (*A. baumannii*). MDR is defined as non-susceptibility to at least one agent in three or more antimicrobial categories (e.g., antipseudomonal cephalosporins, carbapenems, β-lactamase inhibitor combinations, fluoroquinolones, aminoglycosides, polymyxins) [17]. These six leading pathogens are responsible for deaths attributable to bacterial antimicrobial resistance (AMR), rather than overall crude mortality from all infection etiologies [18,19,20,21,22]. *P. aeruginosa* possesses an extraordinary ability to evolve and disseminate antimicrobial resistance in vivo [23,24], due to its genomic complexity, several intrinsic defense mechanisms such as low outer-membrane permeability and antibiotic-efflux pumps, as well as a vast array of virulence genes and biofilm production capacity [25].

*P. aeruginosa* possesses extensive intrinsic and acquired resistance mechanisms; several antipseudomonal agents (e.g., modern β-lactam/β-lactamase inhibitor combinations, cefiderocol, aminoglycosides) remain active depending on regional and institution-specific antibiograms [26]. The World Health Organization recently placed carbapenem-resistant *P. aeruginosa* (CRPA) on its 2024 bacterial priority pathogens list in the high priority category for which the development of new treatment is crucial [27].

Given the marked tendency of *P. aeruginosa* to resist antimicrobial treatments, it is essential to investigate both its emergence and prevalence among patients with UTIs and RTI, as well as its evolving antibiotic resistance trends. In Lebanon, previous studies focused on UTIs caused by *E. coli* [8,28], *K. pneumoniae*, and *P. mirabilis* [7], and limited contemporary epidemiological data exist regarding site-specific *P. aeruginosa* resistance trends in Lebanese secondary/tertiary centers. Moreover, specifically, this study aims to: (1) determine the annual prevalence of *P. aeruginosa* among RTI and UTI patients in a Lebanese tertiary care center over a five-year period; (2) characterize the baseline demographic profiles and specific clinical pathologies associated with these infections; and (3) analyze longitudinal trends in antimicrobial resistance across different antibiotic classes, assessing differences based on patient setting (inpatients vs. outpatients), age, and infection site.

## 2. Materials and Methods

### 2.1. Samples Collection

The study was conducted in the Department of Bacteriology at Sibline Governmental Hospital over a period of five years: 2020, 2021, 2022, 2024 and 2025. This hospital is a public tertiary care referral center located in the Chouf District, Mount Lebanon, with a bed capacity of approximately 100 beds, serving diverse inpatient wards, an intensive care unit (ICU), and outpatient clinics. Data from the year 2023 were excluded due to a major institutional transition and upgrade of the electronic laboratory information system, which resulted in incomplete data logging for that calendar year. Samples were collected from both inpatients and outpatients. Age and sex of the patients were noted. Clean catch urine collection was used for the collection of 2874 samples from outpatients and inpatients, but a Foley was also used for Complete Bed Rest inpatients. For RTI patients, a total of 552 samples were collected from patients. These samples were collected from sputum, bronchial aspirate, bronchoalveolar lavage, bronchial brushings, catheter specimens, bronchial washings and endotracheal tube specimens.

The study size was determined by a convenience sampling strategy, including all eligible clinical isolates of *Pseudomonas aeruginosa* recovered from respiratory and urinary tract specimens at the participating center between January 2020 and December 2025 (5-year non-consecutive surveillance period across a 6-year window (2020–2022, 2024–2025)). No formal a priori sample size calculation was performed; instead, the maximum available sample size within the designated five-year window was utilized to ensure a comprehensive longitudinal analysis of regional prevalence and resistance trends. To minimize selection bias, we implemented strict, uniform inclusion criteria across the entire study period (2020–2022, 2024–2025), extracting data from both hospitalized inpatients and outpatients. To prevent over-representation, only the first isolate per patient per anatomical site (RTI or UTI) within a 30-day window was included, regardless of minor shifts in susceptibility phenotype. Respiratory and urinary isolates were tracked in separate, independent analytical streams. The primary clinical presentations for RTI patients included hospital-acquired pneumonia (HAP), ventilator-associated pneumonia (VAP), exacerbations of chronic obstructive pulmonary disease (COPD), and bronchiectasis. For UTI patients, the predominant pathologies included catheter-associated urinary tract infections (CAUTIs), complicated pyelonephritis, and recurrent cystitis.

The clinical diagnostic criteria for UTI are significant bacteriuria defined as 10^5^ CFU/mL in clean-catch midstream urine or 10^4^ CFU/mL in catheterized urine, accompanied by clinical symptoms (dysuria, fever, flank pain, altered mental status) or systemic inflammatory markers. Asymptomatic bacteriuria and mixed/contaminated cultures (more than 2 species) were excluded. As for RTI, it was evaluated using clinical signs (fever, purulent sputum, new or progressive radiographic infiltrates, leukocytosis) combined with quantitative/semi-quantitative culture thresholds. Sputum specimens were validated using Bartlett’s/Geckler’s criteria (squamous epithelial cells < 10/LPF).

### 2.2. Isolation and Identification of Bacteria

Respiratory and urine samples collected within hospital wards were processed and cultured directly within 2 h of collection. Samples originating from outpatient clinics or delayed transport were preserved in standard sterile transport media (Amies transport medium for respiratory swabs or boric acid preservative containers for urine) and processed within 12–24 h. *P. aeruginosa* isolates were cultured on a complementary set of media (Blood Agar, Chocolate Agar, and MacConkey Agar), each serving a varied diagnostic purpose. Samples were then identified on the basis of standard bacteriology methods: Gram staining and by the API 20 NE gallery system (bioMérieux, Marcy-l’Étoile, France).

### 2.3. Antibiotic Susceptibility Test

Antimicrobial Susceptibility Testing (AST) was conducted using the Kirby–Bauer disk diffusion method, in accordance with Clinical and Laboratory Standards Institute (CLSI) guidelines updated throughout the study period. Direct colony suspension to a 0.5 McFarland standard, inoculation on Mueller–Hinton agar (MHA), incubation at 35 °C for 16–18 h, and manual millimeter measurement of inhibition zones calibrated to annual CLSI M100 standards. The antimicrobial agents commonly prescribed for *P. aeruginosa* were tested (Table 1). Isolates displaying “Intermediate” (I) or “Susceptible-Dose-Dependent” (SDD) categories were analyzed as non-susceptible. *P. aeruginosa* ATCC 27853 was used as a routine QC strain [9].

### 2.4. Statistical Analysis

Individuals’ characteristics were summarized using descriptive statistics. Continuous variables were presented as mean ± standard deviation (± SD), while categorical variables were described as frequencies and percentages. Comparisons between groups were conducted using Pearson’s Chi-square tests and Fisher’s exact test for categorical variables and Student’s *t*-tests and ANOVA for continuous variables. All statistical tests conducted in the study were two-sided, with statistical significance defined as a *p*-value < 0.05. Data collection and analysis were performed using the Statistical Package for Social Sciences (SPSS) software, version 26. To account for multiple simultaneous comparisons across variables and antibiotic families, a Bonferroni correction was applied, adjusting the threshold for statistical significance accordingly to mitigate Type I error risks. Each isolate was counted exactly once toward the total sample size (N = 60 for RTI and N = 38 for UTI) for every demographic variable evaluated. Isolates were categorized at the individual isolate level based on their overall phenotype, ensuring that single isolates tested against multiple antimicrobials were not treated as multiple independent observations. Class-level susceptibility was evaluated at the isolate level; an isolate was classified as non-susceptible to an antibiotic class if it exhibited non-susceptibility to any evaluated agent within that class. Differences in baseline patient demographics (gender, inpatient vs. outpatient setting), collection year, and antimicrobial family susceptibility profiles were compared across isolate phenotypes using Pearson’s Chi-square test or Fisher’s exact test when expected cell counts were <5. Continuous age comparisons between groups were conducted using the two-tailed Student’s *t*-test. Annual linear trend analysis across denominator-adjusted prevalence rates was evaluated using the Chi-square test for trend. Statistical significance was established at a two-sided *p*-value < 0.05.

### 2.5. Ethics Statement

The Research Ethics Committee of the Islamic University of Lebanon provided ethical approval for this study (Reference Number: IUL-EC-26-002, approved on 1 January 2026).

## 3. Results

### 3.1. General and Yearly Prevalence of P. aeruginosa

#### 3.1.1. In Respiratory Tract Infections

**Figure 1:**
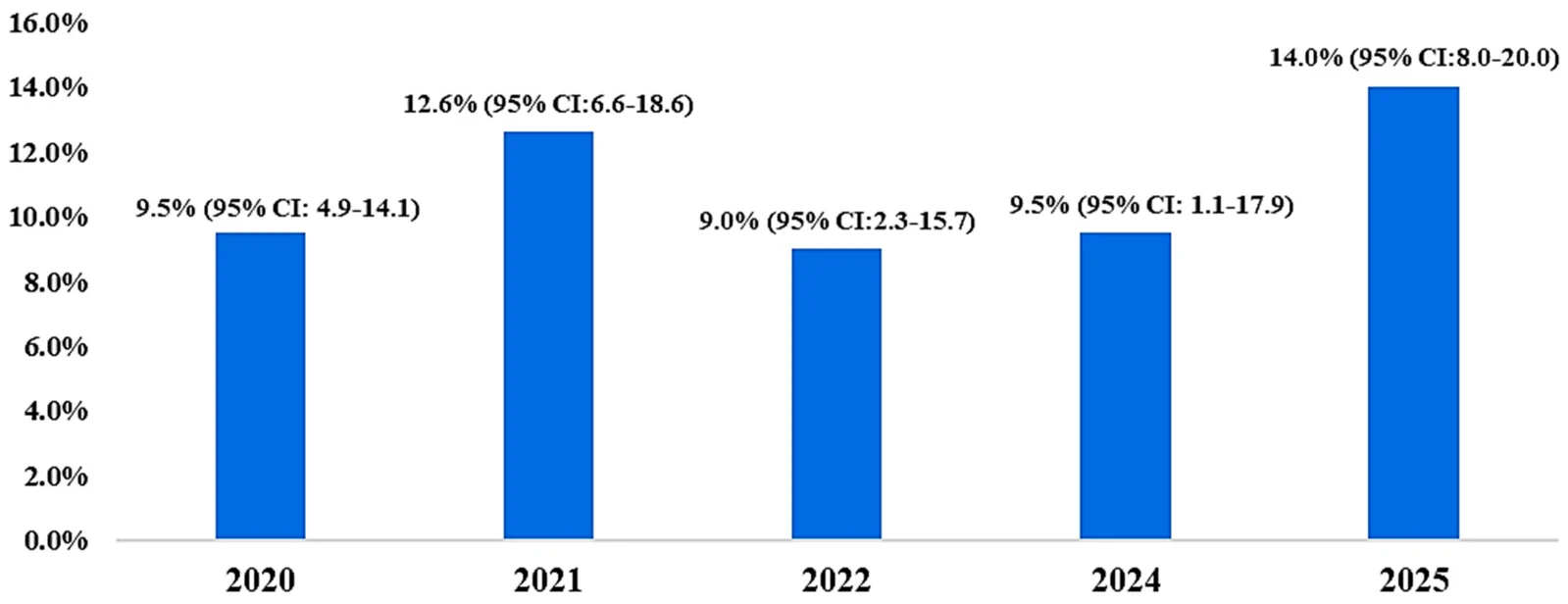
Yearly prevalence of *P. aeruginosa* in respiratory tract infection cases over the years of study. The prevalence fluctuated over the five-year period, hitting a low of 9% in 2022 and peaking at 14% in 2025. Despite these intermediate variations, the overall annual fluctuations demonstrated a progressive increase from 9.5% in 2020 to 14% by the end of the study (CI: confidence interval).

The reported overall prevalence of 10.9% for RTIs (60/552) represents the aggregate prevalence relative to the total number of biological samples analyzed across the entire five-year study period, rather than an unweighted yearly average. This prevalence was not constant over the five-year period, fluctuating between a low of 9% in 2022 and a peak of 14% in 2025. Notably, the annual fluctuations demonstrate a progressive increase from 9.5% in 2020 to 14% in 2025, a variation that was statistically significant (*p* = 0.012), as illustrated in Figure 1.

#### 3.1.2. In Urinary Tract Infections

**Figure 2:**
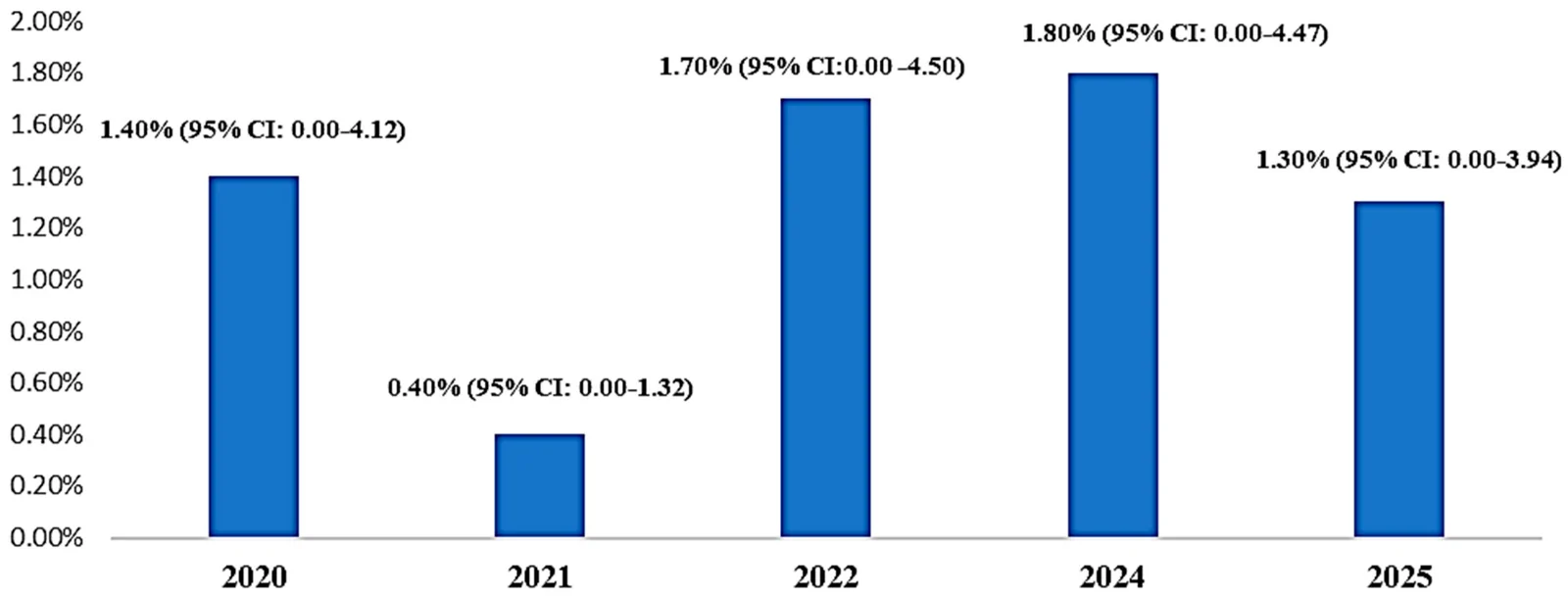
Yearly prevalence of *P. aeruginosa* in urinary tract infection cases over the years of study. Despite minor annual fluctuations—0.4% in 2021 and 1.8% in 2024—the long-term annual fluctuations remained highly stable, shifting only marginally from 1.4% in 2020 to 1.3% in 2025.

The overall prevalence of *P. aeruginosa* in urinary tract infection (UTI) cases throughout the study period was 1.3%, with 38 positive isolates identified among 2874 clinical samples, which represents the aggregate prevalence relative to the total number of biological samples analyzed across the entire five-year study period, rather than an unweighted yearly average. This prevalence was not constant over the five-year period, showing slight fluctuations ranging from a minimum of 0.4% in 2021 to a maximum of 1.8% in 2024. Overall, the annual fluctuations in *P. aeruginosa* prevalence in UTIs between 2020 and 2025 remained relatively stable, with values changing marginally from 1.4% in 2020 to 1.3% in 2025. This variation was not statistically significant (*p* = 0.435), as illustrated in Figure 2.

Annual Numerators and Denominators (n/N) for *P. aeruginosa*: Prevalence for both RTI and UTI is provided in Supplementary Table S1.

It is worth mentioning that for both RTI and UTI, a Chi-square test for linear trend was conducted across yearly denominators to rule out sample volume bias. The proportion of isolates relative to total tests performed remained consistent, demonstrating that yearly prevalence variations reflected genuine epidemiological shifts rather than fluctuations in testing volume.

### 3.2. Demographic Characteristics of Patients Suffering from P. aeruginosa Infections

#### 3.2.1. In Respiratory Tract Infections

Table 1 presents the demographic characteristics (age, sex, and admission status) of patients with RTIs caused by *P. aeruginosa* in the present study. Among *P. aeruginosa*-positive cases, 61.6% occurred in patients aged ≥ 65 years (37 cases), followed by adults at 31.7% (19 cases). In terms of sex distribution, males represented 55% (33 cases), while females accounted for 45% (27 cases). Additionally, the vast majority of patients (93.3%, 56 cases) were admitted to the hospital as inpatients, having hospital-acquired/ventilator-associated pneumonia (46.7%) (Table 2).

#### 3.2.2. In Urinary Tract Infections

Table 2 outlines the demographic characteristics (age, sex, and admission status) of patients with UTIs caused by *P. aeruginosa* in the present study. The majority of positive cases occurred among patients aged ≥ 65 years, accounting for 60.5% (23 cases), followed by adults at 23.7% (9 cases). In terms of sex distribution, males represented 55.3% (21 cases), while females accounted for 44.7% (17 cases). Additionally, most patients (68.4%, 26 cases) were admitted to the hospital as inpatients, with 42.1% suffering from catheter-associated UTI (CAUTI) (Table 3).

### 3.3. Antimicrobial Resistance Profile of P. aeruginosa Isolates

#### 3.3.1. In Respiratory Tract Infections

The detailed susceptibility profiles for the 60 *P. aeruginosa* respiratory isolates, categorized by susceptible (S), intermediate/SDD (I/SDD), resistant (R), and overall non-susceptible (NS = I/SDD + R) status, are presented in Table 4. Across all tested agents, the highest non-susceptibility rate was observed for piperacillin–tazobactam (56.7%, 34/60; comprising 51.7% resistant and 5.0% intermediate isolates), followed by Aztreonam (55.0%, 33/60; 46.7% resistant and 8.3% intermediate) and Ceftazidime (53.3%, 32/60; 46.7% resistant and 6.7% intermediate). Non-susceptibility to Cefepime and Imipenem was recorded at 48.3% (29/60) and 45.0% (27/60), respectively. Conversely, Amikacin demonstrated the highest overall activity, with 83.3% (50/60) of isolates remaining fully susceptible and zero intermediate isolates (16.7% non-susceptible). High susceptibility rates were also retained for Gentamicin (76.7%, 46/60) and Ciprofloxacin (68.3%, 41/60).

**Figure 3:**
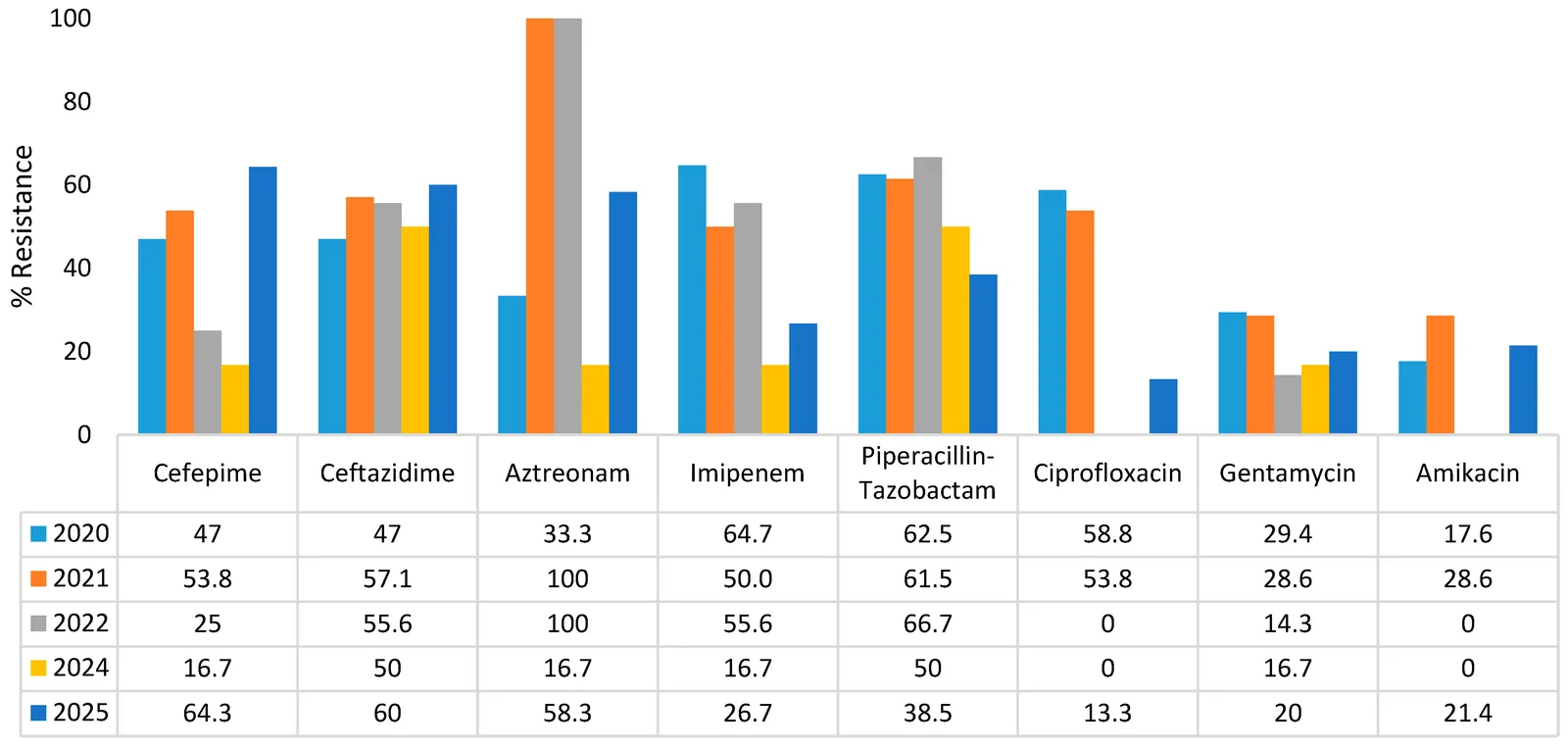
Yearly antibiotic resistance profile of *P. aeruginosa* isolates from RTI cases. Between 2020 and 2025, resistance rates remained relatively stable for Gentamicin and Amikacin, while showing a distinct descending trend for Imipenem, Piperacillin–Tazobactam, and Ciprofloxacin. In contrast, resistance levels followed an ascending trend over the five-year period for Cefepime, Ceftazidime, and Aztreonam.

Figure 3 shows the percentage of resistance of our RTI *P. aeruginosa* to different tested antibiotics in each year of our study period. In 2020, the highest resistance was observed against Imipenem (64.7%) while the lowest resistance was observed against Amikacin (17.6%). In 2021, the highest resistance was observed against Aztreonam (100%) and the lowest was observed against Amikacin and Gentamicin (28.6% for both). In 2022, the highest resistance was observed against Aztreonam (100%) and the lowest was observed against Amikacin and Ciprofloxacin (0% for both). In 2024, the highest resistance was observed against Ceftazidime and Piperacillin–Tazobactam (50% for each) and the lowest was observed against Ciprofloxacin and Amikacin (0% for each). In 2025, the highest resistance profile was observed against Cefepime (64.3%), while the lowest one was observed against Ciprofloxacin (13.3%) (Figure 3).

When focusing on the yearly trend of antimicrobial resistance in those *P. aeruginosa* isolates from RTI patients. Results show that our isolates represent a somewhat stable resistance profile against some antibiotics during different years of study (2020–2025). On the other hand, our isolates show a changeable resistance profile to some other antibiotics (Figure 3).

In the cases of Gentamicin and Amikacin, we did not observe a large difference between the first year (2020) and the last year of study (2025); these changes were registered as 29.4% to 20% for Gentamicin, and 17.6% to 21.4% for Amikacin (Figure 3).

In the cases of Imipenem, Piperacillin–Tazobactam and Ciprofloxacin, the trend was descending between 2020 and 2025. This trend was observed as a decrease from 64.7% to 26.7% for Imipenem, from 62.5% to 38.5% for Piperacillin–Tazobactam and finally, from 58.8% to 13.3% for Ciprofloxacin (Figure 3).

In the cases of Cefepime, Ceftazidime and Aztreonam, the trend of resistance was ascending between 2020 and 2025. This trend was observed as an increase in the percentage of resistance from 47% to 64.3% for Cefepime, from 47% to 60% for Ceftazidime and finally, from 33.3% to 58.3% for Aztreonam (Figure 3).

#### 3.3.2. In Urinary Tract Infections

The antimicrobial susceptibility patterns for the 38 *P. aeruginosa* urinary isolates are summarized in Table 5. Overall, urinary isolates exhibited lower levels of resistance compared to respiratory strains across most antibiotic classes. The highest non-susceptibility rates were observed for Aztreonam and Imipenem (28.9% each, 11/38; both showing 26.3% resistance), followed closely by Ceftazidime (26.3%, 10/38; 21.1% resistant and 5.3% intermediate). Non-susceptibility to Cefepime and piperacillin–tazobactam was identical at 21.1% (8/38; each with 18.4% resistant and 2.6% intermediate isolates). Aminoglycosides showed outstanding activity against urinary isolates: Gentamicin was active against 94.7% (36/38) of strains (5.3% non-susceptible), and Amikacin was active against 92.1% (35/38) of strains (7.9% non-susceptible). Ciprofloxacin also retained high potency, with 81.6% (31/38) of urinary isolates demonstrating full susceptibility.

**Figure 4:**
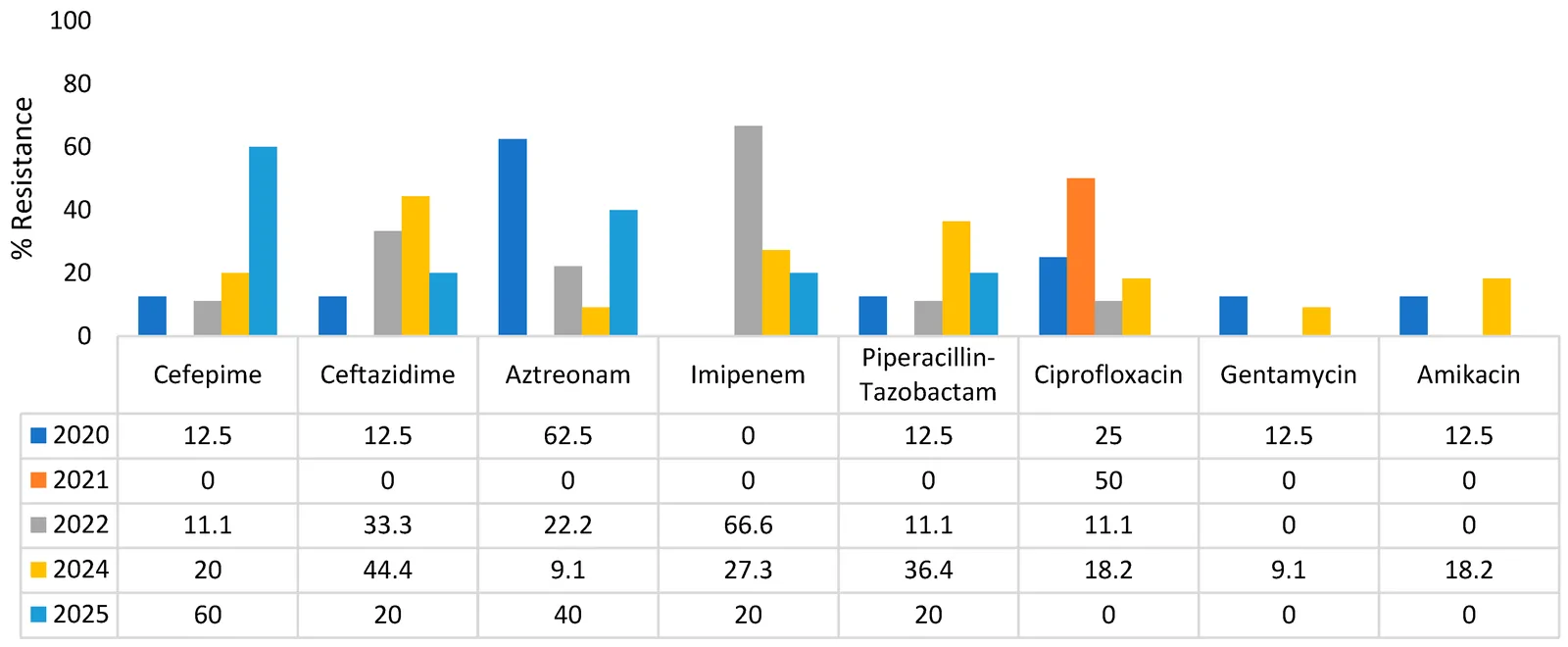
Yearly antibiotic resistance profile of *P. aeruginosa* isolates from UTI cases. Between 2020 and 2025, antibiotic resistance rates showed a descending path for Aztreonam, Ciprofloxacin, Gentamicin, and Amikacin. Conversely, resistance rates followed an ascending path over the same five-year period for Cefepime, Ceftazidime, Imipenem, and Piperacillin–Tazobactam.

Figure 4 shows the percentage of resistance of our UTI *P. aeruginosa* towards different tested antibiotics in each year of our study period. In 2020, the highest resistance was observed against Aztreonam (62.5%), while the lowest resistance was observed against Imipenem (0%). In 2021, the highest resistance was observed against Ciprofloxacin (50%) and it was sensitive against all other tested antibiotics (0% resistance). In 2022, the highest resistance was observed against Imipenem (66.6%) and the lowest was observed against Amikacin and Gentamicin (0% for both). In 2024, the highest resistance was observed against Ceftazidime (44.4% for each) and the lowest was observed against Gentamicin and Aztreonam (9.1% each). In 2025, the highest resistance profile was observed against Cefepime (60%), while the lowest one was observed against Amikacin, Gentamicin and Ciprofloxacin (0% each).

When focusing on the yearly trend of antimicrobial resistance in those *P. aeruginosa* isolates from UTI patients. Results show that our isolates represent a somewhat stable resistance profile against some antibiotics during different years of study (2020–2022, 2024–2025). On the other hand, our isolates show a changeable resistance profile to some other antibiotics (Figure 4).

In the cases of Aztreonam, Ciprofloxacin, Gentamicin and Amikacin, the trend was descending between 2020 and 2025. This trend was observed as a decrease from 62.5% to 40% for Aztreonam, from 25% to 0% for Ciprofloxacin, and finally, from 12.5% to 0% for both Gentamicin and Amikacin (Figure 4).

In the cases of Cefepime, Ceftazidime, Imipenem and Piperacillin–Tazobactam, the trend of resistance was ascending between 2020 and 2025. This trend was observed as an increase in the percentage of resistance from 12.5% to 60% for Cefepime, from 12.5% to 20% for both Ceftazidime and Piperacillin–Tazobactam, and finally, from 0% to 20% for Imipenem (Figure 4).

### 3.4. Demographic Characteristics and Antimicrobial Profiles of Our P. aeruginosa Isolates

#### 3.4.1. In Respiratory Tract Infections

The relationship between patient demographic characteristics, study period, antibiotic classes, and antimicrobial non-susceptibility among respiratory tract *P. aeruginosa* isolates (N = 60) is presented in Table 6. Patient age was significantly associated with non-susceptible phenotypes; patients carrying non-susceptible isolates were significantly older (71.4 ± 18.2 years) than those with fully susceptible isolates (61.6 ± 22.1 years; *p* = 0.028). Admission setting also demonstrated a statistically significant association with non-susceptibility: all non-susceptible respiratory isolates originated from hospitalized inpatients (28/56, 50.0%), whereas zero non-susceptible isolates were recovered from outpatient settings (0/4, 0.0%; *p* = 0.041). Sex distribution did not differ significantly between non-susceptible (48.5% male vs. 44.4% female) and susceptible groups (*p* = 0.598). Likewise, across the study years, non-susceptibility proportions ranged between 33.3% (2022) and 55.6% (2025), but these longitudinal variations did not reach statistical significance at the isolate level (*p* = 0.612).

Across antibiotic classes, aminoglycosides retained the highest level of activity, with only 23.3% (14/60) of isolates demonstrating non-susceptibility compared to 76.7% (46/60) susceptibility (*p* < 0.001). Fluoroquinolone non-susceptibility was also comparatively low (31.7%, 19/60; *p* = 0.004). Conversely, elevated non-susceptibility rates were observed for carbapenems (45.0%, 27/60), cephalosporins (56.7%, 34/60), and beta-lactamase inhibitor combinations (56.7%, 34/60).

#### 3.4.2. In Urinary Tract Infections

The association of clinical factors and antibiotic classes with non-susceptibility among urinary tract *P. aeruginosa* isolates (N = 38) is outlined in Table 7. Sex was significantly associated with antimicrobial non-susceptibility (*p* = 0.046). Male patients exhibited a significantly higher rate of non-susceptible urinary isolates (33.3%, 7/21) compared to female patients (5.9%, 1/17). Patient age was comparable between those with non-susceptible (62.8 ± 25.1 years) and susceptible isolates (63.6 ± 28.1 years; *p* = 0.942). Non-susceptibility was higher among inpatients (26.9%, 7/26) than outpatients (8.3%, 1/12), though this difference did not achieve statistical significance (*p* = 0.231). Longitudinal fluctuations in urinary isolate non-susceptibility across study years (ranging from 0.0% in 2021 to 27.3% in 2024) were also not statistically significant (*p* = 0.785).

Evaluating antibiotic classes at the isolate level revealed significant differences in overall activity. Aminoglycosides demonstrated the highest potency, with only 7.9% (3/38) non-susceptible isolates (*p* < 0.001), followed by fluoroquinolones (18.4% non-susceptible, 7/38; *p* < 0.001) and beta-lactamase inhibitor combinations (21.1% non-susceptible, 8/38; *p* = 0.001). Carbapenem and cephalosporins both exhibited non-susceptibility rates of 28.9% (11/38; *p* = 0.009 for both classes).

## 4. Discussion

Globally, RTIs and UTIs represent the main cause of morbidity, especially in developing countries where these infections still persist among the most frequent clinical presentations requiring intervention. Both types of infections can be symptomatic or asymptomatic, and they can both have negative consequences on infected patients if neglected. This study shows the prevalence and the patterns of antibiotic susceptibility of *P. aeruginosa* isolated from UTI and RTI patients from a tertiary hospital in Lebanon.

According to our data, the overall frequency of *P. aeruginosa* in RTIs was 10.9%, and the majority of *P. aeruginosa* cases in this cohort—61.6%—were in the elderly. The 60 *P. aeruginosa* isolates from RTI cases in the current investigation exhibit distinct patterns of susceptibility to the tested drugs in their general antimicrobial resistance profile. Amikacin, Gentamicin, and Ciprofloxacin showed the highest susceptibility rates (83.3%, 76.7%, and 68.3%, respectively). In contrast, non-susceptibility was highest for Piperacillin–Tazobactam (56.7%), Aztreonam (55.0%), and Ceftazidime (53.3%). Patient age was significantly associated with non-susceptibility, as patients carrying non-susceptible isolates were significantly older (71.4 ± 18.2 years) than those with susceptible isolates (61.6 ± 22.1 years; *p* = 0.028). Additionally, non-susceptibility was significantly associated with admission status (*p* = 0.041), with all non-susceptible isolates recovered from hospitalized inpatients. Sex distribution did not differ significantly between non-susceptible (48.5% male vs. 44.4% female) and susceptible groups (*p* = 0.598). Across antibiotic families, a statistically significant difference in activity was observed (*p* < 0.001), with aminoglycosides retaining the highest overall potency. In order to better understand the incidence of bacterial infections, resistance patterns, and gender-specific characteristics among patients with RTIs admitted to a tertiary-level hospital in South Punjab, Pakistan, Ashiq et al. conducted cohort research. The study’s mean age was 66.5 ± 10.8 years, and 76.4% of the participants were men. The most common Gram-negative bacterium was *P. aeruginosa* (12.89% in males and 15.46% in females). Patients with multidrug-resistant (MDR) infections had a higher death rate (12.22%) than patients with non-resistant strains (4.89%). In ICU patients, resistance to beta-lactams, fluoroquinolones, and macrolides was especially noticeable. Gender-specific differences in bacterial prevalence and resistance patterns were noted, with females exhibiting higher rates of *P. aeruginosa* [29]. Santella et al. conducted another study to ascertain the frequency and pattern of antibiotic susceptibility of bacteria isolated from respiratory samples. Male patients (67.1%) and those between the ages of 40 and 59 (48.6%) had greater rates. *P. aeruginosa* (14.2%) was one of the main bacteria that was shown to be susceptible to Amikacin (85.9%) and resistant to Ciprofloxacin (40.3%) [30]. Behera et al. investigated the frequency and patterns of antibiotic susceptibility of bacteria in intensive care unit patients suffering from lower respiratory tract infections in India. They discovered that 72.72% of male patients and 27.28% of female patients had an infection where *P. aeruginosa* was detected in 7.95% of the cases. Moreover, it exhibited high resistance to cephalosporins (82.18%), but had susceptibility to colistin (88.75%), followed by tigecycline (83.11%), Gentamicin (36.18%), and Amikacin (49.23%) [31].

Concerning UTIs, our findings showed that the general prevalence of *P. aeruginosa* across all evaluated cases was 1.3%, with the majority of patients (60.5%) being elderly (≥65 years). The 38 *P. aeruginosa* isolates recovered from UTI cases displayed distinct susceptibility patterns in their overall antimicrobial profile. Gentamicin, Amikacin, and Ciprofloxacin exhibited the highest susceptibility rates at 94.7%, 92.1%, and 81.6%, respectively. Patient sex was significantly associated with antimicrobial non-susceptibility (*p* = 0.046), with male patients demonstrating a significantly higher rate of non-susceptible isolates (33.3%) compared to female patients (5.9%). Across antibiotic families, significant variations in activity were observed (*p* < 0.05), with aminoglycosides demonstrating the highest overall potency against urinary isolates (92.1% fully susceptible). Gram-negative bacteria accounted for 94% of all isolated strains in 1082 urine bacterial samples, according to a study conducted in Saudi Arabia to examine the epidemiology and antibiotic resistance patterns of urinary tract infections. *P. aeruginosa* (11.55%), *K. pneumonia* (24.58%), and *E. coli* (47.97%) were the most common bacteria linked to UTIs. Patterns of antibiotic resistance revealed that extended-spectrum beta-lactamase (ESBL) was present (30.13%). The most common Gram-negative multidrug-resistant organisms (MDROs) were *E. coli, K. pneumoniae*, and *P. aeruginosa*, which made up 34.42%, 13.95%, and 1.63% of the population, respectively [32]. Another 11-year retrospective analysis on antimicrobial resistance done in Turkey aimed to determine UTIs caused by *P. aeruginosa*. They discovered that 226 (41.8%) of the 540 non-repetitive *P. aeruginosa* strains were identified from male patients and 314 (58.2%) from female patients. The patients’ average age was 66.54 ± 32.62 years. The most successful antimicrobials against *P. aeruginosa* were discovered to be co-trimoxazole and colistin. Third-generation Ceftazidime, Cefoxitin, and Ceftriaxone resistance rates were 48.89%, 89.13%, and 60.37%, respectively; fourth-generation Cefepime resistance was 53.7%; and piperacillin–tazobactam combination resistance was 52.59% [9]. On the other hand, an interesting study performed by Momenah et al. in Saudi Arabia to determine the antimicrobial resistance pattern of *P. aeruginosa* in a tertiary care hospital showed that over the course of 11 years, *P. aeruginosa* in healthcare settings displays increasing levels of resistance to common antimicrobial drugs 5534 of the 61,029 patient specimens—the majority of which were from males over 60—were found to be non-duplicated *P. aeruginosa* clinical isolates. The results of the study showed that colistin had the highest level of antibiotic resistance linked to *P. aeruginosa* isolates (97%), followed by piperacillin/tazobactam (75.8%). Cefepime (42.7%) and Ciprofloxacin (34.3%) had the highest resistance rates among *P. aeruginosa* isolates [33].

Admission status was significantly associated with antimicrobial non-susceptibility in RTIs (*p* = 0.041), with all non-susceptible isolates recovered exclusively from hospitalized inpatients (50.0%, 28/56) compared to zero non-susceptible isolates among outpatients (0.0%, 0/4). This phenomenon is expected, as hospitalized individuals frequently experience prolonged exposure to broad-spectrum antibiotics, cross-contamination via healthcare personnel, and invasive airway interventions (such as endotracheal intubation), which collectively foster a selective environment for multidrug-resistant *P. aeruginosa*.

Our observed broad Levant patterns match wider regional data, where multidrug-resistant (MDR) strains present high critical care resistance but Colistin remains a highly viable last-resort therapeutic choice compared to alternative agents [34]. Furthermore, multi-center documentation in Lebanese clinical settings underscores that the domestic surge in carbapenem resistance among non-fermenters presents heavily in specialized hospital units, heightening public health concerns [35]. Also, a comprehensive epidemiology mapping across the Middle East highlights that isolates from critical care units in Syria exhibit notably high-level resistance to cephalosporins, carbapenems, and aminoglycosides. Meanwhile, surveillance data evaluating multidrug-resistant non-fermenters in Jordan emphasize the necessity of preserving polymyxins [36].

The higher isolation rate in respiratory specimens compared to urinary specimens reflects the inherent tissue tropism of *P. aeruginosa*. It acts primarily as an opportunistic respiratory pathogen in patients with impaired mucociliary clearance or invasive mechanical ventilation. In contrast, it remains a secondary uropathogen compared to *Enterobacteriaceae*. The elevated resistance observed in RTI isolates stems from intense selective pressure exerted by empirical broad-spectrum antibiotic regimens in intensive care units. Furthermore, respiratory isolates frequently form robust biofilms on endotracheal tubes, triggering intrinsic resistance mechanisms. Older patients (≥65 years) carried a significantly higher burden of resistant strains (*p* = 0.01) due to age-related immunosenescence, frequent hospitalizations, and repeated courses of antimicrobials.

On the other hand, when interpreting the longitudinal fluctuations in *P. aeruginosa* prevalence and resistance profiles observed in our cohort, broader contextual factors surrounding healthcare operations during the study period must be considered. The initial surveillance years (2020–2021) coincided with both the peak of the COVID-19 pandemic and a severe national economic crisis in Lebanon. During this phase, tertiary care centers experienced significant disruptions, including altered hospital admission dynamics, heightened critical care bed turnover, and temporary strains on infection prevention and control (IPC) protocols. Furthermore, widespread empirical usage of broad-spectrum antimicrobials in patients presenting with severe acute respiratory illness during the pandemic likely exerted transient selective pressures, contributing to the elevated baseline resistance rates observed for key agents like carbapenems and piperacillin–tazobactam. As institutional workflows stabilized and IPC measures were reinforced in subsequent years (2022–2025), these acute pressures subsided, reflecting a more stabilized endemic baseline for non-fermenting Gram-negative pathogens in the facility.

Finally, a very important point should be highlighted regarding the importance of finding new strategies or techniques to fight the increasing rate of antimicrobial resistance (AMR) in bacteria in general, and, more specifically, in Gram-negative ones. These techniques are numerous, and not limited to the new technique aiming to introduce the antibiotic inside a bacterial cell by a very intelligent strategy known as the “Trojan Horse Technique” [37,38]. As well as the technique aiming to target specific ultra-structures that play an important role in increasing bacterial virulence, such as targeting bacterial “Metallophores” that play an important role in increasing bacterial pathogenicity [19,20,21,22,23,24,25,26,27,28,29,30,31,32,33,34,35,36,37,38,39]. Last, but not least, is the new trend known as “Antimicrobial Bacteriophages,” which aims to use bacteriophages as treatment for infections caused by MDR strains [40,41]. All those strategies can open the window to new solutions regarding the emergence of MDR strains. To address high resistance rates to front-line agents like carbapenems and piperacillin–tazobactam in our Lebanese patient cohort, the “Trojan Horse” strategy uses siderophore–drug conjugates to bypass bacterial membrane barriers and restore antimicrobial delivery. Additionally, lytic bacteriophage therapy offers a novel mechanism to disrupt respiratory and catheter-associated biofilms while re-sensitizing multidrug-resistant *P. aeruginosa* isolates to conventional antibiotics.

## 5. Limitations

First, due to the retrospective nature of the study, the data relies entirely on pre-existing laboratory and electronic health records. Second, our findings are based on data collected from Sibline Governmental Hospital in Lebanon. While the inclusion of both inpatients and outpatients provides a broader perspective than strictly ICU-based studies, the results may not fully represent the entire national epidemiological landscape, and geographic generalizability across different regions of the country should be approached with caution. Third, due to the laboratory-centered nature of the database used, detailed clinical data regarding patient comorbidities and prior antibiotic exposures were unavailable, which limits our ability to correlate resistance patterns with specific clinical risk factors. Additionally, the identification and resistance testing relied entirely on conventional phenotypic methods (API 20 NE and disk diffusion). The lack of molecular confirmation, such as PCR or 16S rRNA sequencing, prevents the characterization of specific resistance genes (e.g., carbapenemases) circulating in this cohort.

## 6. Conclusions

In conclusion, *P. aeruginosa* demonstrates a significantly higher burden and resistance profile in respiratory tract infections compared to urinary tract infections within the studied Lebanese population. The high resistance observed against carbapenems—contrasted with retained susceptibility to aminoglycosides—underscores the critical need for strict antimicrobial stewardship. Elderly and hospitalized patients represent the primary high-risk groups requiring targeted surveillance.

## Figures and Tables

**Table 1:** Antibiotics tested against *P. aeruginosa*.

Antibiotics Groups	Antibiotic
Cephalosporin	Cefepime (30 µg) Ceftazidime (30 µg)
Carbapenem	Imipenem (10 µg)
Beta-lactamase inhibitors	Piperacillin–Tazobactam (100/10 µg)
Fluoroquinolones	Ciprofloxacin (5 µg)
Aminoglycosides	Gentamicin (10 µg) Amikacin (30 µg)
Monobactam	Aztreonam (30 µg)

**Table 2:** Demographic characteristics of RTI patients infected by *P. aeruginosa* (N = 60).

Total Number (%)		
Age	Child (<12) Adolescent (12–18) Adult (19–64) Elderly (≥65)	03 (05) 01 (01.7) 19 (31.7) 37 (61.6)
Gender	Female Male	27 (45) 33 (55)
In–Out Patient	Inpatient Outpatient	56 (93.3) 04 (06.7)

**Table 3:** Incidence of *P. aeruginosa* in UTI according to demographic characteristics (age, gender and in–out patient) (N = 38).

Total Number (%)		
Age	Child (<12) Adolescent (12–18) Adult (19–64) Elderly (≥65)	01 (02.6) 05 (13.2) 09 (23.7) 23 (60.5)
Gender	Female Male	17 (44.7) 21 (55.3)
In–Out Patient	Inpatient Outpatient	26 (68.4) 12 (31.6)

**Table 4:** Antimicrobial Susceptibility Profile of *P. aeruginosa* Isolates from RTI Patients (N = 60).

Antibiotic	Susceptible n (%)	Intermediate/SDD n (%)	Resistant n (%)	Non-Susceptible n (%)
Cefepime	31 (51.7)	3 (5.0)	26 (43.3)	29 (48.3)
Ceftazidime	28 (46.7)	4 (6.7)	28 (46.7)	32 (53.3)
Aztreonam	27 (45.0)	5 (8.3)	28 (46.7)	33 (55.0)
Imipenem	33 (55.0)	2 (3.3)	25 (41.7)	27 (45.0)
Piperacillin–Tazobactam	26 (43.3)	3 (5.0)	31 (51.7)	34 (56.7)
Ciprofloxacin	41 (68.3)	2 (3.3)	17 (28.3)	19 (31.7)
Gentamicin	46 (76.7)	1 (1.7)	13 (21.7)	14 (23.3)
Amikacin	50 (83.3)	0 (0.0)	10 (16.7)	10 (16.7)

**Table 5:** Antimicrobial susceptibility profile of *P. aeruginosa* isolates from UTI patients (N = 38).

Antibiotic	Susceptible n (%)	Intermediate/SDD n (%)	Resistant n (%)	Non-Susceptible n (%)
Cefepime	30 (78.9)	1 (2.6)	7 (18.4)	8 (21.1)
Ceftazidime	28 (73.7)	2 (5.3)	8 (21.1)	10 (26.3)
Aztreonam	27 (71.1)	3 (7.9)	8 (21.1)	11 (28.9)
Imipenem	27 (71.1)	1 (2.6)	10 (26.3)	11 (28.9)
Piperacillin–Tazobactam	30 (78.9)	1 (2.6)	7 (18.4)	8 (21.1)
Ciprofloxacin	31 (81.6)	1 (2.6)	6 (15.8)	7 (18.4)
Gentamicin	36 (94.7)	0 (0.0)	2 (5.3)	2 (5.3)
Amikacin	35 (92.1)	0 (0.0)	3 (7.9)	3 (7.9)

**Table 6:** Antibiotic resistance profile of *P. aeruginosa* isolates from RTI patients by demographic characteristics, admission year, and antibiotic class (N = 60).

Variables	Total Isolates, N (%)	Non-Susceptible Isolates, n (%)	Susceptible Isolates, n (%)	*p*-Value
Age (years)	60 (100)	71.4 ± 18.2	61.6 ± 22.1	0.028 ^a^
Gender				0.598 ^b^
Male	33 (55.0)	16 (48.5)	17 (51.5)	
Female	27 (45.0)	12 (44.4)	15 (55.6)	
Type of Admission				0.041 ^c^
Inpatient	56 (93.3)	28 (50.0)	28 (50.0)	
Outpatient	4 (6.7)	0 (0.0)	4 (100.0)	
Year of Admission				0.612 ^b^
2020	10 (16.7)	5 (50.0)	5 (50.0)	
2021	12 (20.0)	6 (50.0)	6 (50.0)	
2022	9 (15.0)	3 (33.3)	6 (66.7)	
2024	11 (18.3)	4 (36.4)	7 (63.6)	
2025	18 (30.0)	10 (55.6)	8 (44.4)	
Antibiotic Family				
Aminoglycosides	60 (100)	14 (23.3)	46 (76.7)	<0.001 ^b^
Carbapenems (Imipenem)	60 (100)	27 (45.0)	33 (55.0)	0.436 ^b^
Cephalosporins	60 (100)	34 (56.7)	26 (43.3)	0.302 ^b^
Fluoroquinolones (Ciprofloxacin)	60 (100)	19 (31.7)	41 (68.3)	0.004 ^b^
Beta-lactamase inhibitors	60 (100)	34 (56.7)	26 (43.3)	0.302 ^b^

^a^ Student’s *t*-test (Mean ± SD); ^b^ Pearson’s Chi-square test; ^c^ Fisher’s Exact test.

**Table 7:** Antibiotic resistance profile of *P. aeruginosa* isolates from UTI patients by demographic characteristics, admission year, and antibiotic class (N = 38).

Variables	Total Isolates, N (%)	Non-Susceptible Isolates, n (%)	Susceptible Isolates, n (%)	*p*-Value
Age (years)	38 (100)	62.8 ± 25.1	63.6 ±28.1	0.942 ^a^
Gender				0.046 ^c^
Male	21 (55.3)	7 (33.3)	14 (66.7)	
Female	17 (44.7)	1 (5.9)	16 (94.1)	
Type of Admission				0.231 ^c^
Inpatient	26 (68.4)	7 (26.9)	19 (73.1)	
Outpatient	12 (31.6)	1 (8.3)	11 (91.7)	
Year of Admission				0.785 ^c^
2020	8 (21.1)	2 (25.0)	6 (75.0)	
2021	2 (5.3)	0 (0.0)	2 (100.0)	
2022	9 (23.7)	2 (22.2)	7 (77.8)	
2024	11 (28.9)	3 (27.3)	8 (72.7)	
2025	8 (21.1)	1 (12.5)	7 (87.5)	
Antibiotic Family				
Aminoglycosides	38 (100)	3 (7.9)	35 (92.1)	<0.001 ^c^
Carbapenems (Imipenem)	38 (100)	11 (28.9)	27 (71.1)	0.009 ^b^
Cephalosporins	38 (100)	11 (28.9)	27 (71.1)	0.009 ^b^
Fluoroquinolones (Ciprofloxacin)	38 (100)	7 (18.4)	31 (81.6)	<0.001 ^c^
Beta-lactamase inhibitors	38 (100)	8 (21.1)	30 (78.9)	0.001 ^c^

^a^ Student’s *t*-test (Mean ± SD); ^b^ Pearson’s Chi-square test; ^c^ Fisher’s Exact test.

## Data Availability

The original contributions presented in this study are included in the article. Further inquiries can be directed to the corresponding authors.

## Supplementary Materials

The following supporting information can be downloaded at: https://www.mdpi.com/article/10.3390/idr18040087/s1, Table S1: Annual Numerators and Denominators (n/N) for *P. aeruginosa* Prevalence.
