# Supplementary material for: Prevalence and Antimicrobial Resistance of Pseudomonas aeruginosa Recovered from Respiratory and Urinary Specimens in a Lebanese Hospital (2020–2025)
Source: Infect Dis Rep. 2026 Aug 13;18(4):87. doi: 10.3390/idr18040087 (PMC13512891; doi:10.3390/idr18040087)
Supplement: Supplementary file 1 [file idr-18-00087-s001.zip › idr-4429872-supplementary.pdf]

Supplementary Table S1. Annual Numerators and Denominators (n/N) for *P. aeruginosa* Prevalence.

| Year | Respiratory Tract Specimens (n/N, %) | Urinary Tract Specimens (n/N, %) |
|---|---|---|
| **2020** | 10/105 (9.5%) | 8/571 (1.4%) |
| **2021** | 12/95 (12.6%) | 2/500 (0.4%) |
| **2022** | 9/100 (9.0%) | 9/529 (1.7%) |
| **2024** | 11/116 (9.5%) | 11/611 (1.8%) |
| **2025** | 18/136 (13.2%) | 8/615 (1.3%) |
| **Total** | **60/552 (10.9%)** | **38/2874 (1.3%)** |
